# CHALLENGES ASSOCIATED WITH AND LESSONS LEARNED FROM AGGREGATING MEDICAL AID IN DYING DATA IN THE UNITED STATES

**DOI:** 10.1093/geroni/igac059.161

**Published:** 2022-12-20

**Authors:** Molly Nowels, Elissa Kozlov

**Affiliations:** Rutgers School of Public Health, Piscataway, New Jersey, United States; Rutgers School of Public Health, Piscataway, New Jersey, United States

## Abstract

Nine U.S. jurisdictions have legal Medical Aid in Dying (MAID) and produce publicly available data on the use of MAID. We performed a retrospective observational cohort study of MAID data from Oregon, Washington, California, Colorado, Washington DC, Vermont, Hawaii, New Jersey, and Maine. We investigated the total number of deaths from self-administered lethal medications, number of prescriptions for MAID written, characteristics of MAID users and requesters, and challenges of aggregating these data. Over the last 22 years of publicly available data from all states or jurisdictions that have legalized MAID, demographics data was collected for 1,949 people who died by MAID. Persons using MAID were more likely to be non-Hispanic white (95.6%), have some college education (72.2% vs. 26.2%) and a cancer diagnosis (74.0% cancer, 10.9% neurological illness, 15.1% other). Patients who died from MAID prescription ingestion were more likely to have insurance (88.9% insured vs 11.1% uninsured or unknown). Aggregating data came with multiple challenges including states collecting data on different populations (deaths vs prescriptions), with different variable categories, and for different time intervals for reports. More research is needed to better understand why MAID is currently being accessed by an overwhelmingly white, educated, and insured population. This research also highlighted the need for a national research agenda on Medical Aid in Dying. As more states plan to adopt MAID legislation, having standardized data collection elements will help to elucidate how these policies are being implemented and accessed.

